# Therapeutic Advances in Diabetic Kidney Disease

**DOI:** 10.3390/ijms24032803

**Published:** 2023-02-01

**Authors:** Panagiotis I. Georgianos, Vasilios Vaios, Theodoros Eleftheriadis, Evangelos Papachristou, Vassilios Liakopoulos

**Affiliations:** 1 2nd Department of Nephrology, AHEPA Hospital, Aristotle University of Thessaloniki, GR54636 Thessaloniki, Greece; 2 Department of Nephrology, School of Medicine, University of Thessaly, GR41110 Larissa, Greece; 3 Department of Nephrology and Kidney Transplantation, University Hospital of Patras, GR26504 Patras, Greece

**Keywords:** cardiorenal protection, chronic kidney disease, finerenone, SGLT-2 inhibitors, Type 2 diabetes

## Abstract

Although sodium glucose co-transporter type 2 (SGLT-2) inhibitors were initially introduced as glucose-lowering medications, it was later discovered that cardiorenal protection is the most important treatment effect of these agents. A triad of landmark trials consistently showed the benefits of SGLT-2 inhibitors on kidney and cardiovascular outcomes in patients with chronic kidney disease (CKD), irrespective of the presence or absence of Type 2 diabetes (T2D). Furthermore, finerenone is a novel, selective, nonsteroidal mineralocorticoid receptor antagonist (MRA) that safely and effectively improved cardiorenal outcomes in a large Phase 3 clinical trial program that included >13,000 patients with T2D and a wide spectrum of CKD. These two drug categories have shared and distinct mechanisms of action, generating the hypothesis that an overadditive cardiorenal benefit with their combined use may be biologically plausible. In this article, we describe the mechanism of action, and we provide an overview of the evidence for cardiorenal protection with SGLT-2 inhibitors and the nonsteroidal MRA finerenone in patients with CKD associated with T2D.

## 1. Introduction

Type 2 diabetes (T2D) is a metabolic disorder characterized by chronic hyperglycemia caused by disturbances in the action of insulin, insulin secretion, or both [1]. T2D is by far the leading cause of chronic kidney disease (CKD) globally, with ~30–50% of patients with T2D also having reduced kidney function, increased albuminuria, or both [2]. CKD substantially magnifies the risk of adverse cardiorenal outcomes and shortens the life expectancy of patients with T2D [3,4]. The cardiorenal risk increases when the levels of the estimated glomerular filtration rate (eGFR) fall below the threshold of 60 mL/min/1.73 m^2^ [5]. In contrast, the levels of the urinary albumin-to-creatinine ratio (UACR) exhibit a linear risk association with cardiovascular and kidney outcomes without threshold effects [5]. Lifestyle interventions that are recommended by international guidelines for the primary and secondary prevention of cardiovascular disease and CKD in patients with T2D include the restriction of dietary sodium, physical activity, smoking cessation, and weight loss [6,7]. Pharmacological interventions that consist the standard-of-care treatment are adequate glycemic and blood pressure (BP) control, improvement in the serum lipid profile with the use of statins, and regression of albuminuria through the blockade of the renin angiotensin system (RAS) [6,7]. Despite these therapeutic interventions, the residual cardiorenal risk of patients with T2D is very high [2]. Newer therapies to attenuate the progression of CKD and improve cardiovascular outcomes in these high-risk patients are therefore desired.

**Figure 1:**
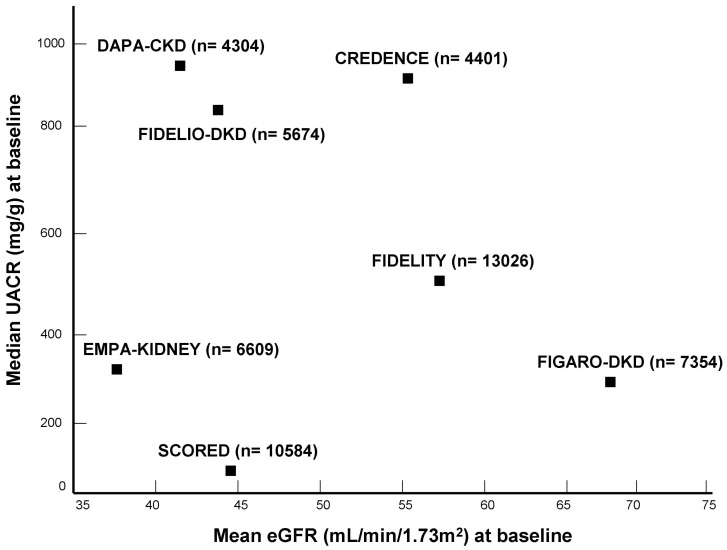
Overview of baseline median UACR and mean eGFR levels of patients enrolled in recent randomized trials that explored the treatment effects of SGLT-2 inhibitors and the nonsteroidal MRA finerenone in CKD associated with T2D.

In this article, we discuss recent advances in the management of CKD associated with T2D, providing an overview of the evidence for cardiorenal protection through the use of sodium glucose co-transporter type 2 (SGLT-2) inhibitors and the nonsteroidal mineralocorticoid receptor antagonist (MRA) finerenone (Figure 1). These two drug categories have complementary mechanisms of action, offering the promise that their combination may provide additive benefits on cardiorenal outcomes as compared with either therapy alone [8]. This crucial research question is currently under investigation in an ongoing clinical trial [9].

## 2. SGLT-2 Inhibitors

SGLT-2 inhibitors were originally approved for improving glycemic control in patients with T2D [10]. However, their initial indication for their use as glucose-lowering medications has been reappraised after the discovery that cardiorenal protection is the main therapeutic effect of SGLT-2 inhibitors [10]. Large Phase 3 clinical trials have demonstrated the impressive benefits of SGLT-2 inhibitors on kidney and cardiovascular outcomes in CKD patients, irrespective of the presence or absence of T2D (Table 1) [11,12,13,14].

In the Canagliflozin and Renal Events in Diabetes with Established Nephropathy Clinical Evaluation (CREDENCE) trial [14], 4401 patients with T2D and CKD were randomized to receive SGLT-2 inhibitor therapy with canagliflozin at a dose of 100 mg/day or placebo. Patients had an eGFR of 30 to <90 mL/min/1.73 m^2^, severely increased albuminuria (UACR of >300 to 5000 mg/g), and were receiving he standard-of-care treatment with maximally tolerated doses of an RAS blocker [14]. The primary outcome of the trial was defined as the combination of the doubling of serum creatinine, end-stage kidney disease (ESKD), or death from renal or cardiovascular causes. CREDENCE was prematurely closed for reasons of efficacy. During a median follow-up of 2.6 years, canagliflozin decreased the risk of the primary outcome by 30% relative to a placebo (hazard ratio (HR): 0.70; 95% confidence interval (CI): 0.59–0.82) [14]. Patients randomly assigned to the canagliflozin group also had a 20% lower risk of cardiovascular death, nonfatal myocardial infarction (MI), or nonfatal stroke (HR: 0.80; 95% CI: 0.67–0.95), as well as a 39% lower risk of hospitalization of heart failure (HF) (HR: 0.61; 95% CI: 0.47–0.80) vs. the placebo [14].

In the Effect of Sotagliflozin on Cardiovascular and Renal Events in Patients with Type 2 Diabetes and Moderate Renal Impairment Who Are at Cardiovascular Risk (SCORED) trial [11], 10,584 patients with T2D and CKD were randomized to double-blinded therapy with sotagliflozin (200–400 mg/day), which is an SGLT-2 inhibitor that also blocks the gastrointestinal SGLT-1 receptor, or a matching placebo. Patients were eligible for this trial if they had an eGFR of 25–60 mL/min/1.73 m^2^ with or without albuminuria and at least one additional cardiovascular risk factor. SCORED was originally designed to explore the non-inferiority of sotagliflozin relative to a placebo in improving ischemic cardiovascular events as well as the superiority of sotagliflozin vs. a placebo with respect to HF events [11]. However, the primary outcome was modified over the course of the trial to the combination of the total number of cardiovascular deaths, hospitalizations for HF, and emergency department visits due to HF. SCORED was stopped early because of a loss of funding, and the median duration of follow-up was only 1.3 years. As compared with the placebo, sotaglifozin reduced the risk of the revised primary outcome by 26% (HR: 0.74; 95% CI: 0.63–0.88) [11]. Despite the lower than planned number of events, sotagliflozin also improved the original co-primary outcome of cardiovascular death, nonfatal MI, or nonfatal stroke (HR: 0.84; 95% CI: 0.72–0.99), as well as the original co-primary outcome of cardiovascular death or hospitalization for HF (HR: 0.77; 95% CI: 0.66–0.91) [11]. However, SCORED was not adequately powered to detect the significant benefits of sotagliflozin on the secondary outcome of progression of CKD (HR: 0.71; 95% CI: 0.46–1.08) [11].

The Dapagliflozin and Prevention of Adverse Outcomes in Chronic Kidney Disease (DAPA-CKD) trial explored the hypothesis that SGLT-2 inhibitor therapy is also effective in preserving kidney function in patients with CKD due to causes other than T2D [12]. Accordingly, 4304 CKD patients with or without T2D were randomized to receive dapagliflozin at a daily dose of 10 mg or a placebo. According to the eligibility criteria, patients had an eGFR of 25–75 mL/min/1.73 m^2^ and moderately to severely increased albuminuria (UACR of 200–5000 mg/g), despite background therapy with the maximally tolerated doses of an angiotensin-converting enzyme inhibitor (ACEI) or an angiotensin receptor blocker (ARB). The primary outcome was defined as the combination of a sustained ≥50% decline in the eGFR after randomization, ESKD, or death from renal or cardiovascular causes [12]. Once again, DAPA-CKD was closed early for reasons of efficacy. During a median follow-up of 2.4 years, the risk of the primary outcome was lower by 39% with dapagliflozin than with the placebo (HR: 0.61; 95% CI: 0.51–0.72) [12]. As compared with the placebo, dapagliflozin also reduced the risk of cardiovascular death or hospitalization for HF by 29% (HR: 0.71; 95% CI: 0.55–0.92) and the risk of all-cause death by 31% (HR: 0.69; 95% CI: 0.53–0.88) [12]. Subgroup analyses showed that the beneficial effects of dapagliflozin on cardiorenal outcomes did not differ between CKD patients with and without T2D [12].

The recently published Study of Heart and Kidney Protection with Empagliflozin (EMPA-KIDNEY) trial randomized 6609 CKD patients with or without T2D to receive empagliflozin at a dose of 10 mg/day or a placebo [13]. EMPA-KIDNEY had wider inclusion criteria: an eGFR of ≥20 to <45 mL/min/1.73 m^2^ or an eGFR of ≥45 to <90 mL/min/1.73 m^2^ with a UACR of ≥200 mg/g. Accordingly, EMPA-KIDNEY was originally designed to include a broader spectrum of patients with CKD, such as those with nonalbuminuric CKD or patients with a lower eGFR, down to the threshold of 20 mL/min/1.73 m^2^. Such patients were under-represented in prior CKD trials with SGLT-2 inhibitors. The primary outcome was the combination of the progression of CKD (defined as ESKD, a sustained decline in the eGFR of <10 mL/min/1.73 m^2^, a sustained decline in the eGFR of ≥40% from the baseline, or death from renal causes) or death from cardiovascular causes [13]. Similar to its predecessors, CREDENCE and DAPA-CKD, the EMPA-KIDNEY trial was stopped early because of the positive results with empagliflozin. During a median follow-up of 2.0 years, empagliflozin provoked a placebo-subtracted relative risk reduction of 28% in the primary composite cardiorenal outcome (HR: 0.72; 95% CI: 0.64–0.82) [13]. This benefit remained unmodified when the patients were stratified into subgroups according to their diabetic status or levels of eGFR at baseline. Furthermore, the risk of hospitalization for any cause was lower in the empagliflozin group than in the placebo group (HR: 0.86; 95% CI: 0.78–0.95), but empagliflozin did not improve the composite outcome of hospitalization for HF or cardiovascular death (HR: 0.84; 95% CI: 0.67–1.07), and did not confer a benefit on survival (HR: 0.87; 95% CI: 0.70–1.08) [13]. The lack of a significant reduction in the relative risk of these secondary outcomes was possibly attributable to the lower than expected number of cardiovascular events because of the premature termination of the trial for reasons of efficacy.

The treatment effect of SGLT-2 inhibitors on kidney and cardiovascular outcomes was quantified in a recent collaborative meta-analysis of 13 large Phase 3 clinical trials involving a total of 90,409 patients (82.7% were patients with T2D and 17.3% were patients without T2D) [15]. In the overall analysis, SGLT-2 inhibitor therapy lowered the risk of CKD progression by 37% relative to the placebo (relative risk (RR): 0.67; 95% CI: 0.59–0.77). This benefit was similar in magnitude among patients with (RR: 0.62; 95% CI: 0.56–0.68) and without T2D (RR: 0.69; 95% CI: 0.57–0.82) [15]. When the analysis was restricted to the four trials that included patients with diabetic kidney disease, the placebo-subtracted reduction in the risk of CKD progression with SGLT-2 inhibitors was 40% (RR: 0.60; 95% CI: 0.53–0.69). When the data for patients with nondiabetic CKD from the DAPA-CKD and EMPA-KIDNEY trials were combined, compared with the placebo, SGLT-2 inhibitors reduced the risk of CKD progression by 30% (RR: 0.70; 95% CI: 0.50–1.00) in patients with ischemic or hypertensive nephropathy, by 40% (RR: 0.60; 95% CI: 0.46–0.78) in patients with glomerular diseases, and by 26% (RR: 0.74; 95% CI: 0.51–1.08) in patients with another or an unknown etiology of CKD [15]. With respect to the treatment’s effect on cardiovascular outcomes, SGLT-2 inhibitors provoked an overall placebo-subtracted reduction of 23% in the risk of cardiovascular death or hospitalization for HF (RR: 0.77; 95% CI: 0.74–0.81) and a placebo-subtracted reduction of 14% in the risk of cardiovascular death (RR: 0.86; 95% CI: 0.51–0.92) [15]. Once again, the cardioprotective benefit of SGLT-2 inhibitors was irrespective of the presence or absence of T2D. These meta-analytic data clearly indicate that the glucose-lowering action is not the sole mechanism through which SGLT-2 inhibitors improve cardiorenal outcomes.

Although the exact mechanistic explanation for the cardiorenal protection afforded by SGLT-2 inhibitors is not yet fully understood, there is a growing body of evidence that these agents exert pleiotropic effects to attenuate the injury to the heart and the kidney [10]. The glycosuric and natriuretic action of SGLT-2 inhibitors has been shown, and resulted in afferent arteriolar constriction and reduced intraglomerular hyperfilitration via restoration of the tubulo-glomerular feedback [16]. Apart from the beneficial actions on intraglomerular hemodynamics, experimental studies have shown that SGLT-2 inhibitors also exert nonhemodynamic actions that include the downregulation of oxidative stress and inflammation as well as improvements in endothelial function [17]. In preclincal models of diabetes, the decreased glucose influx into the proximal tubular cells with SGLT-2 inhibition has been shown to downregulate the generation of reactive oxygen species and suppress the formation of advanced glycation end-products; these actions were accompanied by the amelioration of tubulointerstitial inflammation and fibrosis [18,19]. The anti-inflammatory properties of SGLT-2 inhibitors are also supported by evidence from clinical studies. In the Canagliflozin Treatment and Trial Analysis-Sulfonylurea (CANTATA-SU) trial that enrolled patients with early-stage diabetic kidney disease, the SGLT-2 inhibitor therapy with canagliflozin was effective in reducing the plasma levels of inflammatory biomarkers, such as interleukin-6, matrix metalloproteinase-7, and tumor necrosis factor (TNF) receptor-1 [20]. In the Canagliflozin Cardiovascular Assessment Study (CANVAS), the improvement in cardiovascular outcomes with canagliflozin was shown to be associated with the treatment-induced reduction in the circulating levels of TNF receptor-1 and TNF receptor-2 [21]. Whereas SGLT-2 inhibitors appear to exert potent and direct anti-inflammatory actions, the regression of fibrosis seen with these agents is more likely to be an indirect benefit that is mediated through other actions in the kidney [17]. Evidence to support this hypothesis has been provided by an animal study, in which the genetic knockout of the SGLT-2 in diabetic mice attenuated the development of hyperglycemia and intraglomerular hypertension, but failed to prevent the onset and progression of fibrosis [22].

Additional cardiorenal protective mechanisms have been proposed. For example, the glycosuric action of SGLT-2 inhibitors results in a “fasting-like” state that is characterized by a reduced insulin–glucagon ratio and enhanced ketogenesis [23]. Increased levels of ketone bodies may protect the kidney through the inhibition of mammalian target of rapamycin complex 1 (mTORC1) signaling in tubular cells and podocytes [24]. Furthermore, the increased oxygen consumption for the reabsorption of sodium that is delivered to the distal segments of the tubule acts as a stimulus for increased production of erythropoietin [23]. Apart from the increase in the levels of hematocrit by approximately 3%, the upregulation of hypoxia-inducible factor 2a with SGLT-2 inhibition was also associated with reductions in inflammation and fibrosis, as well as with the stimulation of autophagy at the kidney level [25].

On this basis, the 2022 Kidney Disease: Improving Global Outcomes (KDIGO) guidelines provided a strong Level 1A recommendation that patients with T2D, CKD, and an eGFR of ≥20 mL/min/1.73 m^2^ should receive first-line therapy with an SGLT-2 inhibitor, with the indication of cardiorenal protection [7]. SGLT-2 inhibitor therapy can be continued even if the levels of eGFR fall below the threshold of 20 mL/min/1.73 m^2^, unless this is not tolerated or when the patients initiate renal replacement therapy [7].

## 3. MR Antagonism with Finerenone

Despite receiving standard-of-care treatment with optimal doses of an RAS blocker and the concomitant use of an SGLT-2 inhibitor, patients with T2D and CKD remain at a high risk of adverse cardiorenal outcomes. Evidence from preclinical studies suggested that in such disease states, there is a pathophysiological overactivation of the mineralocorticoid receptor (MR) that leads to the acceleration of end-organ damage through upregulation of the inflammatory and fibrotic pathways [26,27]. Furthermore, a recent analysis from the Chronic Renal Insufficiency Cohort (CRIC) study showed that among patients with diabetic or nondiabetic CKD, increased serum aldosterone levels at baseline are independently associated with an increased long-term risk for the progression of kidney injury to ESKD [28]. Taken together, these data provide mechanistic support to the notion that MR antagonism is another therapeutic opportunity to attenuate the residual cardiorenal risk of patients with T2D (Table 2).

Finerenone is a novel, selective, nonsteroidal MRA that has recently received regulatory approval for improving kidney and cardiovascular outcomes in patients with CKD associated with T2D [31]. The safety and efficacy of finerenone was firstly demonstrated in the Finerenone in Reducing Kidney Failure and Disease Progression in Diabetic Kidney Disease (FIDELIO-DKD) trial [29]. In this trial, 5734 patients with CKD and T2D were randomly assigned to double-blinded therapy with finerenone (10–20 mg/day) or a matching placebo. Eligible patients had to fulfil the following criteria: a UACR of 30 to 300 mg/g, an eGFR of 25 to <60 mL/min/1.73 m^2^ and evidence of diabetic retinopathy, or a UACR of 300 to 5000 mg/g and an eGFR of 25 to <75 mL/min/1.73 m^2^. Therefore, FIDELIO-DKD enrolled patients with T2D and predominantly advanced CKD with severely increased albuminuria. FIDELIO-DKD was originally designed to investigate the treatment effects of finerenone on a primary kidney outcome, defined as the combination of a sustained ≥40% decline in the eGFR from the baseline, ESKD, or death from renal causes [29]. This trial was also powered to detect significant between-group differences in a key secondary cardiovascular outcome, defined as the combination of cardiovascular death, nonfatal MI, nonfatal stroke, or hospitalization for HF. During a median follow-up of 2.6 years, finerenone reduced the risk of the primary kidney outcome by 18% (HR: 0.82; 95% CI: 0.73–0.93) relative to the placebo [29]. In addition, finerenone provoked a placebo-subtracted reduction of 14% in the key secondary composite cardiovascular outcome (HR: 0.86; 95% CI: 0.75–0.99) [29]. Notably, these benefits of finerenone on cardiorenal outcomes were observed in patients who were receiving the optimized standard-of-care treatment with the maximally tolerated doses of an ACEI or an ARB before randomization. With respect to the safety outcomes, the overall incidence of adverse events was balanced between the two groups, with the exception of hyperkalemia-related adverse events, which occurred more commonly with finerenone than with the placebo. However, serious hyperkalemic events with clinical impact were rare. Severe hyperkalemia leading to permanent treatment discontinuation occurred in only 2.3% of the patients assigned to receive finerenone vs. 0.9% of patients assigned to receive the placebo [29].

Whereas finerenone improved the primary outcome by 18% vs. the placebo in the FIDELIO-DKD trial [29], the placebo-subtracted reduction in the relative risk of the primary outcome stimulated by canagliflozin over the course of the CREDENCE trial was 30% [14]. This comparison generated the impression that MR antagonism with finerenone was inferior to SGLT-2 inhibition for improving cardiorenal outcomes in patients with albuminuric CKD and T2D. However, on closer examination, this discrepancy in the reported treatment effects may be attributable to key differences in the design of these two landmark trials. For example, patients with HF with a reduced ejection fraction were eligible in CREDENCE [14], but such patients were excluded a priori from the FIDELIO-DKD trial [29]. In addition, whereas the primary outcome of FIDELIO-DKD was a kidney-specific endpoint that included a sustained ≥40 decline in the eGFR from the baseline [29], CREDENCE had a prespecified cardiorenal composite primary outcome that included a sustained ≥57% decrease in the eGFR from the baseline [14]. A secondary post hoc analysis of the FIDELIO-DKD trial included 4619 out of 5764 participants (81.4% of the overall trial population) who met the CKD eligibility criteria of the CREDENCE trial [32]. Of these, 2291 patients (49.6%) received finerenone, and the remaining 2328 patients (50.4%) were being treated with the placebo. In this “CREDENCE-like” subgroup, as compared with the placebo, finerenone reduced the risk of the composite cardiorenal outcome, which was defined as in the CREDENCE trial, by 26% (HR: 0.74; 95% CI: 0.63–0.87) [32]. Therefore, after adjustment for key differences in their design, both trials showed benefits on cardiorenal outcomes that were similar in magnitude (a 26% reduction in the relative risk with finerenone in FIDELIO-DKD vs. 30% with canagliflozin in CREDENCE).

The safety and efficacy of finerenone in patients with T2D and a broader spectrum of CKD was investigated in the Finerenone in Reducing Cardiovascular Mortality and Morbidity in Diabetic Kidney Disease (FIGARO-DKD) trial [30]. FIGARO-DKD was a complementary Phase 3 trial that included patients with a UACR of 30–300 mg/g and an eGFR of 25–90 mL/min/1.73 m^2^, or patients with a UACR of 300–5000 mg/g and an eGFR of ≥60 mL/min/1.73 m^2^. Accordingly, FIGARO-DKD enrolled patients with less advanced CKD and moderately increased albuminuria; such patients were either excluded or under-represented in the FIDELIO-DKD trial. Unlike FIDELIO-DKD, the primary outcome of FIGARO-DKD was a combined cardiovascular outcome, defined as the time to first occurrence of cardiovascular death, nonfatal MI, nonfatal stroke, or hospitalization for HF [30]. The secondary kidney outcome replicated the primary composite outcome of FIDELIO-DKD. Overall, 7437 patients were randomized to finerenone (10–20 mg/day) or a placebo, on top of background therapy with the maximally tolerated doses of an RAS inhibitor. During a median follow-up of 3.4 years, finerenone reduced the risk of the primary composite cardiovascular outcome relative to the placebo by 13% (HR: 0.87; 95% CI: 0.76–0.98) [30]. The main driver of the cardioprotective benefit of finerenone was the improvement in the risk of hospitalization for HF by 29% (HR: 0.71; 95% CI: 0.56–0.90). Although the composite kidney outcome was not significantly improved, a key secondary kidney-specific outcome that included a sustained ≥ 57% decline in the eGFR from the baseline in the definition occurred less frequently with finerenone than with the placebo (HR: 0.77; 95% CI: 0.60–0.99) [30]. Once again, the incidence of adverse events was comparable between the two study groups. As expected, the proportion of patients who discontinued the trial regimen owing to hyperkalemia was higher in the finerenone group than in the placebo group (incidence rates: 1.2% vs. 0.4%, respectively) [30].

The FIDELITY analysis combined individual patient-level data from the FIDELIO-DKD and FIGARO-DKD trials with the aim of investigating the safety and efficacy of finerenone more precisely relative to the placebo across the whole spectrum of CKD in patients with T2D [33]. Overall, 13,026 patients with a mean eGFR of 57.6 mL/min/1.73 m^2^ and a median UACR of 515 mg/g were included in this combined analysis. Notably, 40% of the patients had an eGFR of ≥60 mL/min/1.73 m^2^ at screening; thus, the identification and recruitment of these patients was based solely on the detection of increased albuminuria. Over a median follow-up of 3.0 years, as compared with the placebo, finerenone reduced the composite cardiovascular outcome of death from cardiovascular causes, nonfatal MI, nonfatal stroke, or hospitalization for HF by 14% (HR: 0.86; 95% CI: 0.78–0.95) [33]. Furthermore, finerenone stimulated a placebo-subtracted reduction in the relative risk of 23% in the composite kidney outcome of a sustained ≥ 57% decline in the eGFR after randomization, ESKD, or death from renal causes (HR: 0.77; 95% CI: 0.67–0.88) [33]. With respect to the safety profile, hyperkalemia leading to permanent withdrawal of the trial regimen occurred more commonly in patients receiving finerenone (1.7%) than in those receiving the placebo (0.6%) [33]. These data indicate that screening for albuminuria is a simple test that would facilitate the identification of patients with T2D who are at high cardiorenal risk in daily clinical practice. Such patients are eligible for add-on therapy with finerenone, which is a pharmacological intervention that confers substantial benefits on long-term cardiovascular and kidney outcomes.

Several mechanisms have been proposed to explain the beneficial impact of finerenone on cardiorenal outcomes. Owing to its short plasma half-life and the lack of active metabolites [34], there is an established impression that the treatment effects of finerenone are largely independent of any reductions in BP. In fact, in the pooled analysis of FIDELITY, the change in the mean systolic BP from the baseline to 4 months of follow-up was only −3.2 mmHg in patients receiving finerenone vs. +0.5 mmHg in patients receiving the placebo [33]. However, a recent subanalysis of the Mineralocorticoid Receptor Antagonist Tolerability Study-Diabetic Nephropathy (ARTS-DN) Phase 2b trial showed that finerenone exerted potent hemodynamic effects, as detected by the gold standard method of 24 h ambulatory BP monitoring in a subset of 240 patients [35]. Between the baseline and Day 90, the placebo-subtracted change in 24 h ambulatory systolic BP was −8.3 mmHg (95% CI: −16.6 to −0.1) with firerenone at 10 mg/day, −11.2 mmHg (95% CI: −18.8 to −3.6) with finerenone at 15 mg/day, and −9.9 mmHg (95% CI: −17.7 to −2.0) with finerenone at 20 mg/day [35]. These data provide a plausible mechanistic explanation for the more frequent occurrence of the adverse events of hypotension, the lower incidence of hypertension, and the early separation of the Kaplan–Meier curves for the composite cardiovascular outcome seen with finerenone over the course of the FIDELIO-DKD and FIGARO-DKD trials [29,30]. Other nonhemodynamic mechanisms may be also involved to explain the delayed separation of the Kaplan–Meier curves for the composite kidney outcome and the sustained benefit of finerenone over the entire duration of follow-up in these two trials [29,30]. For example, evidence from preclinical animal studies supports the notion that the cardiorenal protection afforded by finerenone may be mediated through the suppression of hypertrophic, proinflammatory, and profibrotic pathways as a result of the potent inhibition of MR overactivity with the administration of finerenone [36,37,38,39].

Taking the abovementioned evidence from clinical trials into consideration, the 2022 KDIGO guidelines provided a Level 2A recommendation for the use of a nonsteroidal MRA with proven efficacy in improving cardiorenal outcomes in patients with T2D when the levels of eGFR are ≥25 mL/min/1.73 m^2^, serum potassium levels are within the normal range, and there is persistent albuminuria (UACR of ≥30 mg/g), despite the standard-of-care treatment with maximally tolerated doses of an RAS inhibitor [7]. For the mitigation of the risk of hyperkalemia, the guidelines also recommend the regular monitoring of serum potassium concentrations after the initiation of therapy with a nonsteroidal MRA [7].

## 4. Evidence for a Synergistic Effect with the Combined Use of SGLT-2 Inhibitors and Finerenone

As illustrated above, SGLT-2 inhibitors and the nonsteroidal MRA finerenone are both guideline-directed therapies in patients with CKD associated with T2D [7]. These two drug classes protect the heart and the kidney via independent and complementary mechanistic pathways, thus generating the hypothesis that dual therapy may provide overadditive benefits on cardiorenal outcomes [8]. In a preclinical model of hypertension-induced end-stage organ damage, as compared with either monotherapy, the initial combination of empagliflozin and finerenone at low dosages was more effective in improving functional parameters such as albuminuria and BP, circulating biomarkers such as serum creatinine and uric acid, the histopathological lesions of cardiac and renal fibrosis, and survival in rats [40]. Additional support to the notion that SGLT-2 inhibitors and finerenone may act in a synergistic manner has been provided by post hoc analyses of the outcomes of large trials. For example, a subgroup analysis of the FIDELIO-DKD trial showed that the albuminuria-lowering effect of finerenone was at least as potent in patients receiving SGLT-2 inhibitors as in those who did not [41]. In the FIDELITY analysis, the reduction in the relative risk of cardiorenal outcomes with finerenone was similar in magnitude irrespective of the use of SGLT-2 inhibitors as a background therapy [42]. Similarly, in the DAPA-CKD trial, the benefit of dapagliflozin on kidney and cardiovascular outcomes was consistent in patients who were or were not treated with a steroidal MRA at baseline [43]. Combination therapy may be also attractive not only for enhanced efficacy but also for reasons of safety, since the risk of hyperkalemia associated with the use of MRAs appears to be mitigated by the potassium-lowering action of SGLT-2 inhibitors [44]. Subgroup analyses showed fewer adverse events of hyperkalemia in finerenone-treated patients than in those not receiving SGLT-2 inhibitors at baseline [41,42].

These preliminary data provided the rationale for the design of randomized clinical trials aiming to directly investigate the potential superiority of a combination therapy with an SGLT-2 inhibitor and an MRA over each respective monotherapy. The Rotation for Optimal Targeting of Albuminuria and Treatment Evaluation-3 (ROTATE-3) was an open-label, randomized, crossover trial that explored the albuminuria-lowering effects of the SGLT-2 inhibitor dapagliflozin (10 mg/day) and the steroidal MRA eplerenone (50 mg/day), administered either individually or in combination, in 48 CKD patients with or without T2D [45]. Short-term treatment for 4 weeks with the combination of dapagliflozin and eplerenone was associated with a clinically meaningful reduction of 53% in the UACR, an albuminuria-lowering effect that was greater than that conferred by either agent alone. Furthermore, a signal of improved safety was observed, since the incidence of hyperkalemia was lower with the dual therapy than with eplerenone alone [45].

The long-term safety and additive efficacy of the combination therapy is currently under investigation in the ongoing Combination Effect of Finerenone and Empagliflozin in Participants with CKD and Type 2 Diabetes Using a UACR Endpoint (CONFIDENCE) trial [9]. CONFIDENCE is a Phase 2 double-blind, randomized, three-armed, parallel group trial that is planning to recruit 807 patients with T2D, Stage 2–3 CKD, and severely increased albuminuria (UACR of ≥300 to <5000 mg/g), who are receiving the standard-of-care treatment with maximally tolerated doses of an ACEI or an ARB [9]. These patients will be randomly assigned to combination therapy with finerenone (10–20 mg/day) plus empagliflozin (10 mg/day), or monotherapy either with empagliflozin (10 mg/day) or with finerenone (10–20 mg/day) for 6 months. The primary outcome is defined as the difference among groups in the proportional change in UACR from the baseline to the study’s end. Changes in the eGFR and treatment-induced hyperkalemia will be also assessed as secondary safety endpoints [9]. If this trial provides evidence for an overadditive albuminuria-lowering effect through the combined use of finerenone and empagliflozin that is not counteracted by an excess risk of adverse events, then simultaneous initiation of an SGLT-2 inhibitor and finerenone could become the roadmap for maximal cardiorenal protection in patients with CKD and T2D in the future.

## 5. Conclusions and Perspectives

For almost 18 years, the standard-of-care treatment for patients with CKD associated with T2D included only improving the control of hyperglycemia and hypertension, as well as the regression of albuminuria through the administration of RAS blockers. After this long period of hiatus, there is now a new era in the pharmacological treatment of diabetic kidney disease. On the basis of strong evidence from clinical trials, the inhibition of SGLT-2 and antagonism of MR with finerenone are two guideline-directed therapeutic interventions used to attenuate the progression of CKD and improve cardiovascular morbidity and mortality in this high-risk group of patients [6,7]. These two drug classes have shared and distinct mechanisms of action, and preliminary evidence suggests that their combination may confer overadditive cardiorenal benefits as compared with either therapy alone. A conclusive answer to this important research question is expected by the ongoing CONFIDENCE trial [9]. Additional therapeutic options for diabetic kidney disease may become available in the upcoming future. The ongoing Effect of Semaglutide vs. Placebo on the Progression of Renal Impairment in Subjects with Type 2 Diabetes and Chronic Kidney Disease (FLOW) trial is currently investigating whether the glucagon-like peptide-1 receptor agonist (GLP-1RA) semaglutide is superior to a placebo in improving kidney outcomes in a population of 3534 patients with CKD and T2D at a high risk of progression in kidney disease [46]. The FLOW trial is expected to be completed in late 2024.

## Figures and Tables

**Table 1:** The effect of SGLT-2 inhibitors on kidney and cardiovascular outcomes in randomized trials conducted in CKD patients with or without T2D.

Trial	Size, *n*	Key Eligibility Criteria	Proportion with T2D, *n* (%)	Median Follow-Up, Years	Key Outcomes
CREDENCE [14] (canagliflozin, 100 mg/day, vs. placebo)	4401	T2D and CKD with an eGFR of 30–90 mL/min/1.73 m^2^ and a UACR of 300–5000 mg/g Stable maximally tolerated doses of an RAS blocker Excluded patients with suspected nondiabetic CKD	4401 (100%)	2.6	Doubling of serum creatinine, ESKD, or death from CV or renal causes: HR = 0.70; 95% CI: 0.59–0.82. Death from CV causes, nonfatal myocardial infarction, or stroke: HR = 0.80; 95% CI: 0.67–0.95. Hospitalization for HF: HR = 0.61; 95% CI: 0.47–0.80.
SCORED [11] (sotagliflozin, 200–400 mg/day, vs. placebo)	10,584	T2D and CKD with an eGFR of 25–60 mL/min/1.73 m^2^ At least 1 additional risk factor for CV disease	10,584 (100%)	1.3	Death from CV causes, hospitalization for HF, or emergency visits for HF: HR = 0.74; 95% CI: 0.63–0.88. Progression of CKD ^§^: HR = 0.71; 95% CI: 0.46–1.08.
DAPA-CKD [12] (dapagliflozin, 10 mg/day, vs. placebo)	4304	CKD with an eGFR 25–75 mL/min/1.73 m^2^ and a UACR of 200–5000 mg/g. Stable maximally tolerated doses of an RAS blocker. Excluded polycystic kidney disease, lupus nephritis, or anti-neutrophil cytoplasmic antibody-associated vasculitis	2906 (68%)	2.4	Sustained ≥50% decrease in eGFR, ESKD, or death from renal and CV causes: HR = 0.61; 95% CI: 0.51–0.72. Death from CV causes or hospitalization for HF: HR = 0.71; 95% CI: 0.55–0.92. All-cause mortality: HR = 0.69; 95% CI: 0.53–0.88.
EMPA-KIDNEY [13] (empagliflozin, 10 mg/day, vs. placebo)	6609	eGFR 20–45 mL/min/1.73 m^2^ or eGFR 45–90 mL/min/1.73 m^2^ with a UACR of ≥200 mg/g. Clinically appropriate dose of an RAS blocker, unless not indicated or not tolerated. Excluced patients with polycystic kidney disease.	3040 (46.0%)	2.0	Progression of CKD ^†^ or death from CV causes: HR = 0.72; 95% CI: 0.64–0.82. All-cause hospitalizations: HR = 0.86; 95% CI: 0.78–0.95. Hospitalization for HF or death from CV causes: HR: 0.84; 95% CI: 0.67–1.07.

Abbreviations: CKD, chronic kidney disease; CI, confidence interval; CV, cardiovascular; eGFR, estimated glomerular filtration rate; ESKD, end-stage kidney disease; HF, heart failure; HR, hazard ratio; RAS, renin angiotensin system; T2D, Type 2 diabetes; UACR, urinary albumin-to-creatinine ratio. ^§^ The progression of CKD in SCORED was defined as a sustained decrease of ≥50% in the eGFR from the baseline for ≥30 days, long-term dialysis, renal transplantation, or a sustained eGFR of <15 mL/min/1.73 m^2^ for ≥30 days; ^†^ The progression of CKD in EMPA-KIDNEY was defined as ESKD, a sustained decrease in eGFR to <10 mL/min/1.73 m^2^, a sustained decrease in eGFR of ≥40% from the baseline, or death from renal causes.

**Table 2:** Cardiovascular and kidney outcome trials with the nonsteroidal MRA finerenone in patients with CKD and T2D.

Trial	Size, *n*	Key Eligibility Criteria	Median Follow-Up, Years	Key Outcomes
FIDELIO-DKD [29] (finerenone, 10–20 mg/day, vs. placebo)	5734	T2D and CKD with a UACR of 30 to 300 mg/g, an eGFR of 25 to <60 mL/min/1.73 m^2^, and diabetic retinopathy T2D and CKD with a UACR of 300 to 5000 mg/g and an eGFR of 25 to <75 mL/min/1.73 m^2^ Optimized background therapy with maximally tolerated doses of an RAS blocker Excluded patients with HF with a reduced ejection fraction	2.6	Kidney failure, a sustained ≥40% decline in the eGFR from the baseline, or death from renal causes: HR = 0.82; 95% CI: 0.73–0.93. Kidney failure, a sustained ≥57% decline in the eGFR from the baseline, or death from renal causes: HR: 0.76; 95% CI: 0.68–0.90. Death from CV causes, nonfatal myocardial infarction, nonfatal stroke, or hospitalization for HF: HR = 0.86; 95% CI: 0.75–0.99.
FIGARO-DKD [30] (finerenone, 10–20 mg/day, vs. placebo)	7434	T2D and CKD with a UACR of 30 to 300 mg/g and an eGFR of 25 to 90 mL/min/1.73 m^2^ T2D and CKD with a UACR of 300 to 5000 mg/g and an eGFR of ≥60 mL/min/1.73 m. Optimized background therapy with maximally tolerated doses of an RAS blocker. Excluded patients with HF with a reduced ejection fraction	3.4	Death from CV causes, nonfatal myocardial infarction, nonfatal stroke, or hospitalization for HF: HR = 0.87; 95% CI: 0.76–0.98. Kidney failure, a sustained ≥40% decline in the eGFR from the baseline, or death from renal causes: HR = 0.87; 95% CI: 0.76–1.01. Kidney failure, a sustained ≥57% decline in the eGFR from the baseline, or death from renal causes: HR: 0.77; 95% CI: 0.60–0.99.

Abbreviations: CKD, chronic kidney disease; CI, confidence interval; CV, cardiovascular; eGFR, estimated glomerular filtration rate; HF, heart failure; HR, hazard ratio; RAS, renin angiotensin system; T2D, Type 2 diabetes; UACR, urinary albumin to creatinine ratio.

## Data Availability

Not applicable.
